# Validity of mid-upper arm circumference in assessing thinness among older children aged 5–9 years: a cross-sectional study

**DOI:** 10.1186/s12887-025-06120-7

**Published:** 2025-10-03

**Authors:** Terry Namirembe, Ezekiel Mupere, Teddy Namubiru, Peter James Elyanu, Nicolette Nabukeera-Barungi

**Affiliations:** 1 Department of Paediatrics and Child Health, Makerere University College of Health Sciences, P.O. Box 7072, Kampala, Uganda; 2 Baylor College of Medicine Foundation-Uganda, Kampala, Uganda

**Keywords:** Body mass index, Mid-upper arm circumference, Thinness, Older children, Validity

## Abstract

**Background:**

With the high burden of malnutrition, accurate yet convenient anthropometric measurements are vital. Although body mass index (BMI) for age and sex z score (BAZ) is the recommended assessment method for children aged 5–9 years, it is quite cumbersome. Uganda adopted use of the convenient mid-upper arm circumference (MUAC) for these children. However, the basis for MUAC use in this age-group is weak, requiring more evidence generation. We therefore determined validity of MUAC in assessing thinness among children aged 5–9 years.

**Methods:**

This was a cross-sectional study. Demographic and anthropometric data was taken from children aged 5–9 years who attended 3 health facilities in Kampala district. MUAC was measured using colour-coded MUAC tapes for children aged 5–9 years and compared with the BAZ. We used a MUAC cut-off of 145 mm for wasting and below 140 mm for severe wasting as per the national guidelines. The sensitivity, specificity, negative and positive predictive values of MUAC were determined using BAZ as the gold standard. Receiver Operating Characteristic curves were plotted to determine the validity of MUAC.

**Results:**

Of the 767 children, 51.5% were female and the mean age was 7.2 +/-1.6 years. According to MUAC, 1% had severe acute malnutrition (SAM), 2.7% moderate acute malnutrition (MAM) and 96.2% had no acute malnutrition. The sensitivity and specificity of MUAC in assessment of acute malnutrition (SAM and MAM) were 22.7%, 95% CI (11.5–37.8) and 97.3%, 95% CI (95.9–98.4) respectively. The positive and negative predictive values were 34.5%, 95% CI (17.9–54.3) and 95.4%, 95% CI (93.6–96.8) respectively. The area under the ROC curve was 0.30, 95%CI (0.206–0.394), indicating MUAC was a poor assessment tool for thinness, even after stratifying for age and Human Immune-deficiency Virus status.

**Conclusion:**

Despite MUAC being convenient, it showed a low diagnostic performance in assessing thinness among children 5–9 years. This reiterates the need for more research on the correct MUAC cut-offs for this age-group.

**Supplementary Information:**

The online version contains supplementary material available at 10.1186/s12887-025-06120-7.

## Background

The focus of malnutrition has always been on children below 5 years of age. This is because of the high burden of malnutrition in this age group, where 148.1 million (22.3%) were stunted, 45 million were wasted and 37 million (5,6%) were overweight in 2022 [1]. However, children aged 5 years and above also have poor nutrition indicators. In 2022, among children and adolescents aged 5–19 years, 390 million were overweight, including 160 million who were living with obesity. Another 190 million were living with thinness (body mass index (BMI)-for-age more than two standard deviations below the reference median) [2]. With these large numbers, there is need to effectively screen for malnutrition in these children. Unfortunately, current global targets do not explicitly capture data on this important age group of children and adolescents (aged 5–19 years), despite representing key groups of the population that are particularly burdened by poor diets and resulting malnutrition [3]. This lack of focus has led to a distinct lack of information about the nutritional status of the children above 5 years.

Globally, there has been a reduction in thinness for children 5–9 years to 8.9% and 10.9% for girls and boys respectively by 2019. However, Africa showed increasing levels of under- nutrition from 181 million to 222 million undernourished children while obesity and overweight doubled from 2006 to 2016. Particularly, the lowest mean BMI in children aged 5–9 years was seen in East Africa in both sexes [4]. This calls for more efforts to accurately screen and guide efforts to reduce the burden of malnutrition in this age group. In order to achieve this, accurate and convenient screening methods have to be available.

While using anthropometric measurements, the weight for height Z score charts and MUAC tapes are put in colours, with the red being severe wasting and yellow implying moderate wasting while green means no wasting, and includes the overweight children [5]. MUAC tapes therefore have obesity in the green colour-code yet it is a form of malnutrition. On the other hand, Body Mass Index (BMI) for age and sex z-scores (BAZ) cater for obesity. BAZ is the World Health Organisation (WHO) recommended standard indicator for nutritional status assessment among children aged five years and above [5, 6]. However, BAZ requires use of multiple instruments like a stadiometer, accurate weighing scales and growth standard charts. These materials may not be readily available and require regular maintenance. This makes it field unfriendly and time consuming in busy outpatient departments as well as community settings. It also requires more skilled personnel like trained health workers. This is unlike Mid-Upper Arm Circumference (MUAC) which is easy to use even by community extension workers and caregivers of children. In addition, the instruments are easy to use in the field. This convenience created a demand for development of a MUAC tape for older children.

MUAC has been used since the 1960 s to assess nutritional status for children under 5 years, particularly as a proxy for muscle mass, which is a key component of lean body mass. MUAC measures the arm muscle and fat area using a colour-coded tape measure with a fixed cut off for moderate and severe wasting. It has also been found to be a predictor of morbidity and mortality [7–9]. Correlation of MUAC to Weight for Height (WFH) has been extensively studied for children under five [10–12]. Of late, MUAC is increasingly being used with children over 5 years and adolescents, mostly because it offers convenience. Whereas one needs to take the weight, height, calculate BMI and then use a chart to determine the BAZ, MUAC only requires a simple portable tape where the measurement and classification is made on wrapping the tape around the mid-upper arm. Although there is promise about the value of its use in older children [13], more research is needed to establish the cutoff measurements for specific age ranges. Because of lack of evidence, there are no WHO cut-off points provided for children above 5 years and there were no universal internationally accepted MUAC cutoffs for children and adolescents. Despite this, some countries have established their own cutoffs [14]. In Uganda, MUAC tapes are provided by the United Nations Children’s Fund (UNICEF) and are used for assessment of thinness among children above 5 years. This is incorporated into the national guidelines [15]. The MUAC cut offs used in these guidelines are based on Valid International standards after a study done in Syria with support from UNICEF Syria [16]. The cut offs by age for severe wasting are less than 14 cm, 16 cm and 20 cm for 5–9 years, 10–14 years, 15–17 years respectively. These tapes are colour-coded like into red, yellow and green representing severe wasting, moderate wasting and no wasting respectively.

While using MUAC to assess for malnutrition in children above 5 years in clinical practice in Uganda, anecdotal reports indicate that some children are misclassified by MUAC when compared to BAZ which is the gold standard for determining thinness at this age according to WHO. In addition, a study in Uganda found that children above 5 years took longer to recover from thinness compared to the younger ones in an HIV program [17]. The slow recovery could be due to the anthropometric methods used in this age group. This observation has led to a need to study the accuracy of MUAC for children above 5 years so that the children are not missed or over diagnosed and unnecessarily kept in nutritional programs. This study therefore aimed at determining usefulness of MUAC in assessing thinness among children aged 5–9 years.

## Methods and materials

### Study design and setting

This was a cross-sectional study. The study involved 3 health facilities in Kampala, namely Baylor-Uganda clinic, Kawaala and Kisenyi health centres. The facilities are all located in the capital city of Uganda. Baylor-Uganda is a Non-Governmental Organization involved in care for Human Immune-deficiency Virus(HIV)-infected children and their families. This large outpatient clinic has a total of 7000 children in care and on an average serves 270 children between 5 and 9 years in a month. We selected an HIV program because we needed to include a proportion of children with malnutrition in the age-group of 5–9 years, and these tend to concentrate in HIV clinics. We could not assess validity of MUAC if the sample did not have children with thinness. Kisenyi health Centre IV on the other hand attracts an average of 400 children aged 5–9 years in a month. Kawaala health Centre III serves an average of 200 patients aged 5–9 years in a month. The later attend to stable outpatients with minor ailments. The rationale for using multiple centres was to get the large sample size in the required time.

### Study participants

The study population included 782 children aged 5–9 years that came to the above-mentioned health centres with their caretakers between the period of October to December 2021. Only those whose caretakers gave written informed consent were enrolled. We excluded 25 children for whom it was impossible to accurately measure anthropometric measurements. To estimate the appropriate sample size to determine the sensitivity and specificity of MUAC, the modified Kish Leslie formula was employed (Leslie 1985). We used the specificity and sensitivity found in a study conducted in the same setting but using MUAC for children below 5 years and published in 2020 [18]. The sample size was 767 children.

Proportionate sampling was used to determine the proportion of participants from each study site. These were calculated to be 353 children from Kisenyi, 176 from Kawaala and 238 from Baylor-Uganda clinic. Then at each site; we used consecutive sampling to choose the participants who met the study criteria until we achieved the required number of participants per site.

### Study procedure

A pre-tested questionnaire was administered to each of the caretakers by the 10 research assistants who were nutritionists or the principal investigator. The anthropometric indicators measured were height, weight, and MUAC. Height of the children was taken using a stadiometer with a vertical back board, a fixed base and movable head board (Infant/Child Shorr-Board^®^, Maryland). The stadiometer was placed on a levelled floor. The children were required to take off their shoes and hair ornaments stand erect with their heels, head, shoulders and buttocks against the stadiometer wall. Weight (kg) was measured to the closest 0.1 kg using a digital scale (Seca 813 Hamburg, Germany) with children in light clothing. MUAC was measured using the colour-coded tape for the age 5–9 years [16]. The tapes were placed at the exact midpoint (between the acromion and olecranon) of the flexed left upper arm. The reading was taken when the arm was hanging down the body and the tape fitted not too loose but also not too tight on the skin. It was measured to the nearest millimetre. Each of the measurements was taken thrice and the average was considered. Body Mass Index was calculated by dividing the average weight in kilograms by the average height in meters squared. The final BMI was rounded off to the nearest 0.1 kg/m2 and then used WHO reference charts 2007 to determine the BMI for age z-scores. Using these charts, children were considered normal if their BAZ was − 2SD to + 1SD, thinness if −2SD to −3SD and severe thinness if <−3SD. Thinness according to MUAC was considered to be less than 145 mm. Each observer was assigned to take one particular measurement for the whole study to reduce inter observer bias.

### Statistical analysis

All questionnaires were checked for accuracy and completeness before leaving the field. These were stored under lock and key in a safe place. Data was entered into Epi-data V3.02. It was later exported into STATA V14 where it was cleaned and analysed. The primary outcome was thinness which was defined as less than − 2SD according to BAZ and less than 145 mm according to MUAC. Normal BAZ is (−2SD to + 1SD), thinness (−2SD to −3SD) and severe thinness(<−3SD). We estimated the sensitivity, specificity, positive and negative predictive values with BAZ as the gold standard. After that, we compared the prevalence of malnutrition using BMI and MUAC using kappa statistic. Later, Receiver Operating Characteristc curves (ROC) were used by plotting the true positive rate (sensitivity) against the false positive rate (1-specificity). The area under the ROC curves gave the accuracy of the MUAC values (optimal cut offs) in assessing nutritional status. We then estimated the positive and negative predictive values. At determining the optimal cut off, the number of participants taken by MUAC to be having acute malnutrition and those by BMI was then determined. BMI being the gold standard, the Positive Predictive Value was obtained by getting the percentage of participants who actually had malnutrition, of those who were listed by MUAC as having malnutrition. The Negative Predictive Value was be obtained by getting the percentage of those who actually did not have malnutrition of those who were listed by MUAC as not having malnutrition. These were repeated after stratification for age and HIV status.

## Results

We analysed data for 767 children and of these, about half were female; 395 (51.5%) with a mean age of 7.2 years, standard deviation of 1.6years. Majority of the caretakers were females 660 (86.1%) between ages of 25–34 years 320 (41.7%). It was also noted that most of the children had their mother as the caretaker 498 (64.9%) as shown in Table 1.

**Table 1:** Sociodemographic characteristics of children aged 5-9 years and caregivers at outpatient clinics of selected health facilities in Kampala

**Variable**		**Frequency** **(n=767)**	**Percentage**
Child characteristics			
	Age in years (mean ± SD)	(7 ± 1.6)	
Age in years	5 years	172	22.4
	6 years	141	18.4
	7 years	137	17.9
	8 years	115	15.0
	9 years	202	26.3
Gender	Male	372	48.5
	Female	395	51.5
Region of residence	Central	497	64.8
	Western	119	15.5
	Eastern	79	10.3
	Northern	36	4.7
	None Ugandan	36	4.7
Care giver characteristics			
Age (years)	Mean ± SD	33.41 ± 9.74	
Age group	<18	34	4.4
	18-24	85	11.1
	25-34	320	41.7
	35-44	234	30.5
	>=45	94	12.3
Sex	Male	107	14.0
	Female	660	86.1
Relationship with child	Mother	498	64.9
	Father	75	9.8
	Sibling	76	9.9
	other	118	15.4
Level of education	Primary	200	26.1
	Secondary	366	47.7
	Tertiary	129	16.8
	None	72	9.4
Monthly income	(median, IQR)	64US$, (0-107US$)	
Anthropometric categorization for the participants			
BMI for age	Normal (−2SD to +1SD)	622	81.1
	Thinness (−2SD to −3SD)	25	3.3
	Severe thinness(<−3SD)	19	2.5
	Overweight (+1SD to +2SD)	65	8.5
	Obesity(>2SD)	36	4.7
MUAC Categories	Median >14.5cm	738	96.2
	MAM 14.0cm - 14.5cm	21	2.7
	SAM <14.0cm	8	1.0

### Validity of mid-upper arm circumference

The sensitivity of MUAC in determining thinness (MAM and SAM) was 22.7%, 95% CI (11.5–37.8). The specificity of MUAC was 97.3%, 95% CI (95.9–98.4). On stratification between the younger and older children, sensitivity was better for the children aged 5–7 years at 29.6% and much lower for the older children 8–9 years at 11.6%. The positive predictive value of MUAC was 34.5%, 95% CI (17.9–54.3). The negative predictive value of MUAC was 95.4%, 95% CI (93.6–96.8) as shown in Table 2 below.

**Table 2:** Validity of MUAC as an assessment tool for thinness among children aged 5–9 years at outpatient clinics of selected health facilities in Kampala

Validity		Overall		5 to 7 years		8 to 9 years	
		%	95%CI	%	95%CI	%	95%CI
Sensitivity	Pr (+|D)	22.7	11.5–37.8	29.6	13.8–50.2	11.8	1.5–36.4
Specificity	Pr (-|~D)	97.3	95.9–98.4	96.2	93.9–97.8	99.0	97.1–99.8
PPV	Pr (D|+)	34.5	17.9–54.3	33.3	15.6–55.3	40.0	5.3–85.3
NPV	Pr (~ D|-)	95.4	93.6–96.8	95.5	93.1–97.3	95.2	92.2–97.3
Prevalence	Pr(D)	5.7	4.2–7.6	6.0	4.0-8.6	5.4	3.2–8.4
Accuracy		93.1	91.1–94.8	92.2	89.3–94.5	94.3	91.2–96.6

*PPV * Positive Predictive value, *NPV* Negative Predictive value

**Fig. 1:**
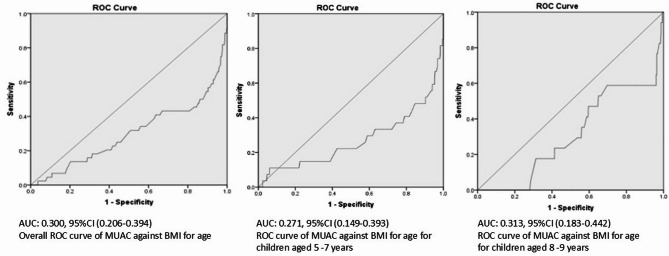
ROC curve of MUAC against BMI for age among children by age-group

The area under the curve was 0.30, 95%CI (0.206–0.394) in the ROC curve in Fig. 1 below. This pattern did not change after stratification based on age as shown in Fig. 1.

### Validity of MUAC as an assessment tool for thinness in the children with HIV compared to those without HIV

The sensitivity of MUAC in children with HIV and those without was low at 27.8%, 95% CI (9.7–53.5) and 19.2%, 95% CI (6.6–39.4) respectively.

The specificity of MUAC was 97.1%, 95% CI (94.1–98.8) among HIV positive children. Similarly, the specificity of MUAC remained high at 97.2%, 95% CI (95.7–98.7) among the children without HIV.

**Fig. 2:**
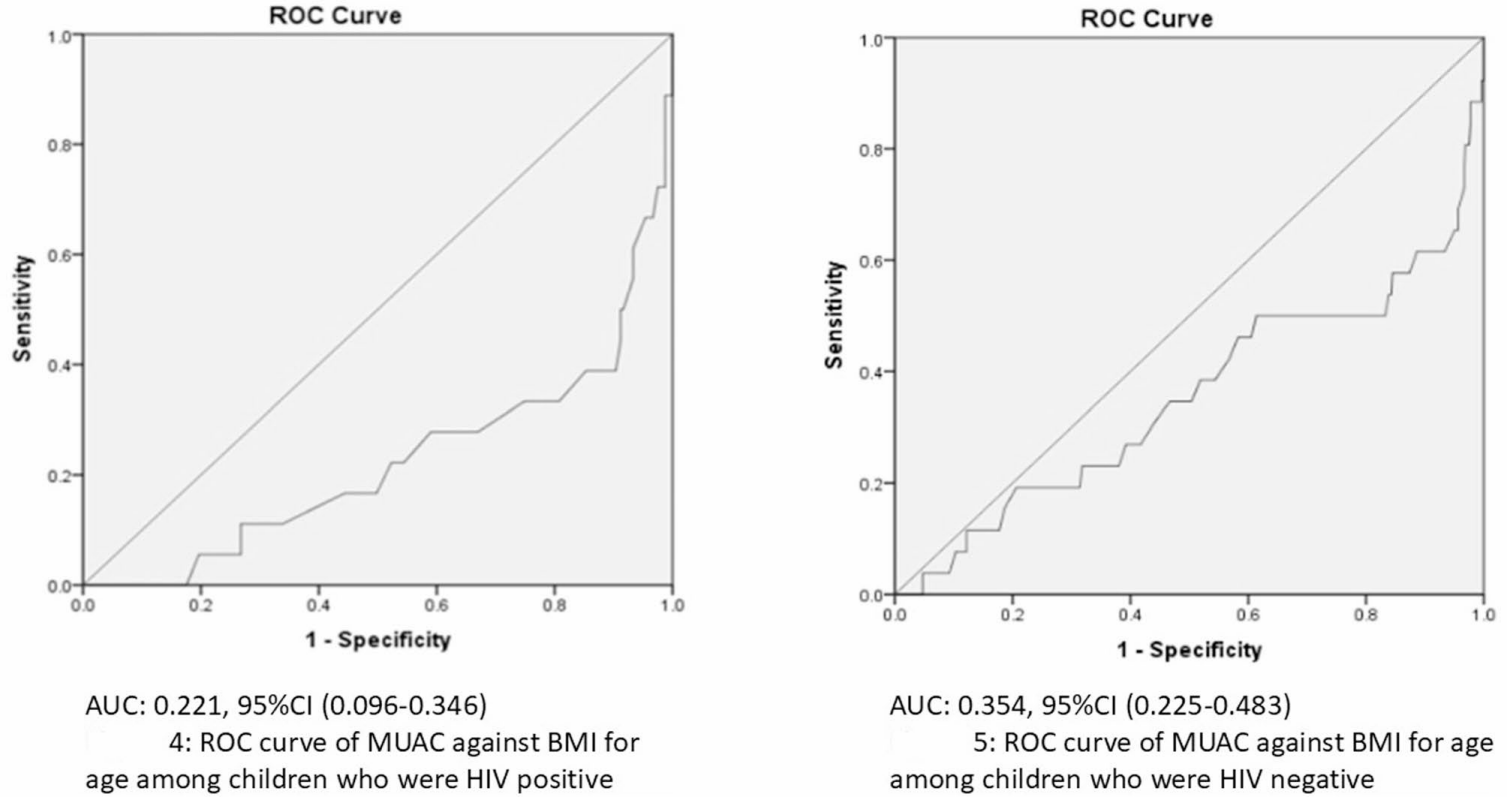
ROC curve of MUAC against BMI for age among children by HIV status

Figure 2 below show area under the curve of 0.22 and 0.35 among the children with and without HIV respectively. This implies that in both the HIV and non-HIV population, MUAC is a poor assessment tool for thinness among children 5–9 years (Table 3).

**Table 3:** Validity of MUAC as an assessment tool for thinness based on HIV status among children aged 5–9 years at outpatient clinics of selected health facilities in Kampala

Performance		HIV positive		HIV negative	
		%	95%CI	%	95%CI
Sensitivity	Pr (+|D)	27.8	9.7–53.5	19.2	6.6–39.4
Specificity	Pr (-|~D)	97.1	94.1–98.8	97.5	95.7–98.7
PPV	Pr (D|+)	41.7	15.2–72.3	29.4	10.3–56.0
NPV	Pr (~ D|-)	94.7	91.1–97.1	95.7	93.6–97.3
Prevalence	Pr(D)	7.0	4.2–10.8	5.1	3.4–7.4
Accuracy		92.2	88.2–95.2	93.5	91.0-95.5

## Discussion

This could be the first cross-sectional study that aimed at determining the validity of MUAC in assessing thinness of children 5–9 years in Uganda.

With BAZ as the gold standard for assessment of thinness, sensitivity of MUAC was found to be very low at 22.7%, 95% CI (11.5–37.8) with a high specificity. After stratification by age and HIV status, this pattern did not change much. This implies that MUAC can hardly identify children with thinness but can easily identify those who don’t have thinness. The positive and negative predictive values were 34.5%and 95.4% respectively and this pattern was similar after stratification for age and HIV status. This signifies that the chances of subjects to truly test positive for thinness using MUAC are low but high for truly testing negative. The discrepancy could be due to the low prevalence of thinness in the community given prevalence influences the negative and positive predictive values.

Although studies done to examine validity of MUAC in this age group are few, results are similar showing wide range sensitivities but consistently high specificity. For example, a study done among children aged 5–10 years in Sri Lanka, showed moderate correlation between MUAC and thinness with sensitivity of 69% [19]. This could be because they used a much higher MUAC cut off of 167 cm that is way above the cut off we used in our study. The mean age was similar at 7 years, but the prevalence of thinness in that study was not reported. This further points to the fact that MUAC is not a good tool for assessing thinness in this age group.

Among the age group of 5–9 years, the increase in mid-upper arm circumference is very minimal, with a smaller standard deviation of about 0.8–0.9 [20]. For this reason, it would be thought that a single MUAC cut off point would be even more appropriate for assessing thinness. However, due to increasing heterogeneity of muscle mass among children in this age group, the mid-upper arm circumference is highly varied among children with same BMI [21].

Validity of MUAC could be age dependent in that it decreases with increasing age. For example, a study done in Mulago among children of 6–59 months showed that MUAC had a relatively higher sensitivity of 47.5% at the WHO standard cut off of 12.5 cm [18]. However, when further divided between the younger age group and older ones, the sensitivity was relatively better with younger children compared to the older ones. Similarly in this study, when further classified, MUAC sensitivity was better in the younger ones compared to the older ones. This may suggest that sensitivity of MUAC declines as children grow older. This is because muscle mass heterogeneity increases as children grow older as children grow older.

Also, typically in this age group, children usually increase more in non-fat mass compared to fat mass, however, due to increasing prevalence of obesity, fat mass is increasing. This fat may be deposited in the gynoid and android areas rather than the mid-upper arm [21]. Hence an increasing discrepancy between the mid-upper arm circumference and the nutritional status. Hence, BAZ will classify them as well-nourished but MUAC will classify them as thin. It’s important to note that this study was conducted in Kampala the capital of Uganda where it’s postulated to have the double burden of malnutrition. In fact, in this study, overweight and obesity were actually more prevalent compared thinness which correlates to the above theory that increasing prevalence of overnutrition, is leading to decreasing validity of MUAC. Although thinness was slightly higher among children with HIV, MUAC was similarly a poor tool in assessing thinness in this population. Keeping MUAC in the national guidelines with the current cut-off points will misclassify children’s nutritional status and keep them unnecessarily longer in the nutrition program. This would lead to wastage of the already limited resources.

The strength of this study that it was a large study with a sample size of 767 participants. In addition, the study was done in outpatient clinics where children had minor ailments hence the participants are a fair representation of the community. This is probably the first study in our setting to determine validity of MUAC in assessing thinness in this age group yet it has been used for a while. The study limitation was that we did not assess for dehydration which could affect the weight of the children but not the MUAC. However, being stable outpatients, it is unlikely that many children were dehydrated. The potential lipodystrophy among children with HIV could affect both the MUAC and BAZ measurements, due to HIV or the antiretroviral drugs, but none of the children were receiving stavudine and zidovudine which are the biggest causes of lipodystrophy. In addition, it would have been better to use a community study but there were no resources to conduct a large community study. Multiple people doing measurements at same time, could lead to inter observational discrepancies. However, we ensured that the same research assistant took one anthropometric measurement consistently and they had weekly training and supervision. The analysis could have been better if we controlled for more variables that affect weight and height.

In conclusion, MUAC can hardly identify children aged 5–9 years with thinness but it is good at ruling it out. Findings suggest MUAC is poor at assessing thinness among children 5–9 years and hence researchers should consider studies using different cut-off points for thinness in order to improve its validity. The new guidelines should consider the BAZ scores other than MUAC where there is discrepancy between the two measurements.

## Supplementary Information

Supplementary Material 1.

## Data Availability

Data will be available on request from the corresponding author.

## Abbreviations

BAZ: Body Mass Index for Age Z-Score
BMI: Body Mass Index
HIV: Human Immuno-deficiency Virus
IMAM: Integrated Management of Acute Malnutrition
KCCA: Kampala Capital City Authority
MoH: Ministry of Health
MUAC: Mid-Upper Arm Circumference
NPV: Negative Predictive Value
PPV: Positive Predictive Value
ROC: Receiver Operating Characteristic curves
W/A: Weight-for-Age
WFL/H: Weight for Length/Height
WHO: World Health Organization
WHZ: Weight for Height Z score
