# Supplementary material for: Validity of mid-upper arm circumference in assessing thinness among older children aged 5–9 years: a cross-sectional study
Source: BMC Pediatr. 2025 Oct 3;25:762. doi: 10.1186/s12887-025-06120-7 (PMC12495605; doi:10.1186/s12887-025-06120-7)
Supplement: Supplementary file 1 — Supplementary Material 1. [file 12887_2025_6120_MOESM1_ESM.pdf]

APPENDICES

APPENDIX ONE: QUESTIONNAIRE TO INVESTIGATE VALIDITY OF MUAC IN ASSESSING THINNESS AMONG CHILDREN WITH ANDWITHOUT HIV AGED 5-9 YEARS- AMULTICENTER STUDY

Investigator ID: ______________________________

Participant initials : ________________

Study ID NO____________   Date of interview: _____/_____/_____

CHILD DEMOGRAPHICS

Date of birth (dd/mm/year)	
Age	……….years ………..months
Sex	1. Male<br>2. Female
Religion	
Tribe	
Family size	Adults …………..<br><br>Children …………
Birth interval	Number of siblings older than participant…………<br><br>Number of siblings younger than participant……..

CAREGIVER DEMOGRAPHICS

Age in years	
Sex	1. Male<br>2. female
Nationality	
Religion	1. Christian<br>2. Muslim

	3. Other; specify……………………………..
Relationship to child	
Level of education	1. Primary<br>2. Secondary<br>3. Tertiary<br>4. None
Occupation	
Monthly income	
Distance from nearest health facility	

Household characteristics

Water source	1. Tap water<br>2. Borehole<br>3. Spring or well<br>4. Other
Hand washing after toilet use	1. YES<br>2. NO
Housing	1. Semi-permanent<br>2. Permanent
Does household have a latrine/toilet?	1. Yes<br>2. No
How many households share this toilet/latrine	1. 1<br>2. 2<br>3. 3<br>4. >3<br>5. not sure

Medical History

Fever in the last 2 weeks	1. YES<br>2. NO
Diarrhea in last 2 weeks	1. YES<br>2. NO
Cough in the last 2 weeks	1. YES<br>2. NO<br><br>If YES; duration of cough<br>1. < 2 weeks<br>2. >2 weeks
Any chronic illnesses	1. HIV<br>2. Congenital heart disease<br>3. Sickle cell<br>4. Asthma<br>5. Others; specify……………..
History of treatment for malnutrition before age 5 years	1. YES<br>2. NO
Currently on RUTF supplementation	1. YES<br>2. NO
Number of hospitalizations in last 6 months	

For children with HIV;

Are you on HIV medication?	1. Yes<br>**2.** No
If yes how long?	
Number of hospitalizations in last 6 months	

Food security

Are you worried about your next meal?	1. Yes<br>2. No
Main source of food (tick all that apply)	1. Grown by family<br>2. Bought<br>3. Other; specify…………………………………..
If grown by family; what is the main food grown?	
For those who buy food; food item most commonly bought	

Nutritional history

Daily meal	1. YES<br>2. NO
Number of meals per day	
Food eaten yesterday	Breakfast……………………….<br>Lunch…………………………..<br>Supper……………………………<br>Other…………………………….
How many times have you had meat or milk (for the children) in last week?	

Anthropometry

Weight	
Height	
MUAC	
